# Abstract of Proceedings of the Buffalo Medical Association

**Published:** 1865-12

**Authors:** F. M. Johnson

**Affiliations:** Sec’y.


ART. III.—*Abstract of Proceedings of the Buffalo Medical Association.*

Nov. 8th, 1865.

The Association met at 7½ o'clock, the President, Dr. Ring, in the chair. Present, Drs. Ring, Strong, Congar, Cronyn, Whitney, Gay, Rochester, Miner and Johnson.

The report of the last meeting was read and accepted.

Dr. Cronyn observed that he was not sorry to have given the Secretary the trouble of reading the minutes of the case reported by Dr. Congar, for it seemed to him that an explanation more satisfactory than any given for the immediate cause of "Hemiplegia," in this case should be sought; that the hemorrhage, of itself, would have been sufficient to produce an epileptic attack, and a very temporary paralysis, there is no doubt, but it would be general and of so short duration that the mere loss of blood, and its shock could have but little to do in the continuance of an Hemiplegia of week's duration. Had at present two cases of "Hemiplegia" in females, occurring in one as the result of an altercation with a neighbor; the power of utterance was partially lost, and partial loss of power in both arm and leg of right side. The other is the result of an epileptic attack. It was attended with entire loss of consciousness for about thirty hours, which, on passing off, left her unable to articulate, with complete hemiplegia of right side. She is slowly recovering her power of speech, but not of side. A case more analagous to the one read, occurred to him last summer, in the person of a young woman in her first confinement; as the head pressed upon the perineum, nothing was anticipated but an easy and speedy delivery. She had a violent convulsion—made application of forceps immediately—and the supposed cause of the irritation removed—there was no return of convulsion, comparatively little loss of blood; the next day there seemed nothing remarkable about the case, save that she complained of her right arm and leg being sore, and to some degree powerless. This was attributed by those around to her efforts at pulling and pushing, during a portion of her labor; but a day or two put an end to such excuse, for it was found that she was completely paralyzed on that side, and the subject of acute "Cerebritis," of which she died within ten days. These cases afford ample opportunity for consideration, each in an apparent state of health before attack, yet each becoming suddenly paralyzed under entirely different circumstances, with evidently the same pathalogical derangement, or change in the cerebro-spinal centres, and which may be explained, very probably, by adopting the different features accredited to the theory of embolism.

He related the case of a boy fourteen years of age, who received a pistol shot accidentally, better than three weeks since. The ball entered the left orbital plate of the frontal bone, and passed directly backwards through the brain, on the plane of entrance, some four and a half inches, where it could be felt by the probe; blood and brain substance escaped freely through the wound. The boy fell, as if dead. He was taken up unconscious, and remained so for some forty-eight hours. His right arm and leg were found paralyzed, and his left eye very much swollen; his head was ordered to be shaved, and an evaporizing lotion applied to eye and to head, if it became hot; indeed, the directions were to wait and to watch. Consciousness was gradually restored. The right leg has regained much of its power, but not the arm. The external wound was healed, and now, at nearly four weeks, he sits up, eats, and complains of nothing, nor is there anything unusual or observable in his mental condition, save that he sometimes forgets that he asked for a thing, when it is brought to him. This is certainly an interesting case, and involves many questions in cerebral pathology.

Dr. GAY would state, in this connection, that in the case of Mr. Waite, of this city, who was accidentally shot with a revolver, that the ball entered the cranium in the right temporal region, and passed into the brain. At first he appeared wholly unconscious, but after five or six hours he became fully conscious, and said that at the time of his apparent unconsciousness he knew perfectly well what was going on, and understood what was being said, but was unable to articulate; could not speak or move much. Dr. Gay did not design to report the case, but only to speak of it in this connection, and call attention to the fact that this consciouness still remained.

Dr. MINER remarked that he was sorry to be obliged to modify, in any way, Dr. Gay's report of Mr. Wait's condition. He had just left Mr. Wait's bedside; he found him this evening unconscious, breathing stertorously, and evidently near his end. His appeararance of apparent convalescence had continued up to 1 or 2 o'clock that afternoon. During ten days preceding, his attending physician, Dr. Stork, reported him as having been cheerful and hopeful, with no symptom of danger except some headache; had laughed and joked with his visitors and family, with his usual good feeling.

Dr. ROCHESTER reported an extremely rare dislocation of the patella, as follows: On the evening of the 28th of August, a large and growing lad, of sixteen years, fell, striking his knee on the curb-stone. On seeing him, a few minutes after the accident, he found the righ patella completely dislocated outward, and *turned upon its edge*, standing at right angles with its normal position.

The knee was slightly swollen, and very tender and painful to the touch. Chloroform was given to complete anæsthesia, and two or three unavailing efforts were made to replace the bone, the limb being fully extended, and the foot elevated. Dr. Gay, who was also present, then, by a different proceedure, at once reduced the dislocation; he flexed the leg upon the thigh, and the thigh upon the body, and then extended, and at the same time rotated the limb, pressing, meantime, firmly upon the patella, which immediately slipped into its place, while the limb was passing from flexion to extension. Dr. Rochester was glad to put so rare a case upon Gay the deserved credit of success in the reduction of a dislocation hardly ever met with, and which, when encountered had sometimes baffled some of Europe's most distinguished surgeons.

Dr. Gay wished to call the attention of the association to the use of the sesquichloride ferri, in diphtheria and diphtheritic sore throat. He had, during the last few months, prescribed it frequently in diphtheritic disease, with excellent results. Had made use of it in a solution, consisting of xx grs. of this salt to an ℥ of glycerine, to be given in drachm doses.

Dr. Rochester remarked that the use of a solution of sesqui chloride ferri, in the treatment of diphtheritic disease was not new to the profession in this city; that he and many other members of the profession both here and elsewhere had used it during the last two years. He had used it both as a local and constitutional remedy, and could speak very highly of it as a remedy in the class of cases mentioned.

Dr. Gay said that he was not aware that this preparation of iron had been used by the profession in this city. He had made use of it on recommendation of a medical friend residing in a neighboring city, and supposed its use new to the profession generally. He believes diphtheria to be a blood disease, and that it should be treated as such, and that local remedies are of little or no use.

Dr. Strong remarked that he believed diphtheria should be treated as such. He had treated several cases with preparations of iron and quinine, with marked good effects, and believes that an intelligent use of proper remedies should always be resorted to.

Dr. Miner said he thought this and other forms of iron had been extensively used, both locally and constitutionally, and that they all possessed at least the merit of not doing any great harm, provided the application to the throat was made with a soft brush, and the sponge and swab avoided. That any application to diphtheritic exudation, either prevented its extension or in any way abridged the duration of the disease, was not probable, and indeed it would seem almost certain that it, like other blood diseases, could not be greatly influenced in its termination by medication. The chief indications appeared to be, as in scarlet fever and other diseases, where blood poison is supposed to constitute the malady, to sustain the strength by all available means, while very little dependence could be placed upon medicine in producing any important modification in the disease itself.

F. M. Johnson, Sec'y.

